# The attitudes about life-sustaining treatment among cardiac surgery ICU patients and their families

**DOI:** 10.3389/fsurg.2023.1079337

**Published:** 2023-05-19

**Authors:** Si Sun, Hao Zhang, XiaoYan Xiong

**Affiliations:** ^1^ Department of Nursing, Nanjing First Hospital, Nanjing Medical University, Nanjing City, China; ^2^ Department of Intensive Care Unit, Nanjing First Hospital, Nanjing Medical University, Nanjing City, China

**Keywords:** life-sustaining treatment, attitude, cardiac surgery ICU, patients, family members

## Abstract

**Purpose:**

To investigate the attitudes among cardiac surgery ICU patients and their families regarding life-sustaining treatment.

**Methods:**

A total of 172 pairs of patients in the cardiac surgery ICU of Nanjing First Hospital and their family members were enrolled in this study that examined their attitudes toward life-sustaining treatment using a willingness to care for life-sustaining treatment questionnaire. The consistency of the attitudes of patients and family members toward life-sustaining treatment was analyzed by the chi-square test with a paired design.

**Results:**

The most popular life-sustaining treatment for cardiac ICU patients was noninvasive mechanical ventilation (79.1%); the most unpopular was intra-aortic balloon counterpulsation (48.3%). Most patients and their families had not considered electric defibrillation (65.7%), but most understood and were willing to permit cardiopulmonary resuscitation (76.2%). Few family members agreed that patients should receive a pacemaker (25.0%). The consistency of life support attitudes of patients and their families ranged from 12.8% to 60.5% for procedures both would agree to, 1.2% to 19.8% for procedures they were unwilling to permit, and 0.6% to 39.0% for procedures they had not considered. Kappa values ranged from 0.218 to 0.597 (*P* < 0.05), with general consistency.

**Conclusion:**

Cardiac surgery ICU patients families are generally consistent in their attitudes toward life-sustaining treatment, and family members’ choices are not representative of patients’ wishes.

## 1. Introduction

Life-sustaining treatment (LST) is required for the care of critically ill patients. LST is a medical treatment used to maintain the function of major organs or to treat a disease or injury and is meant to prolong a patient's life. LST can prolong a patient's life, but not all of the damage done can be reversed, and it is a medical treatment that consumes many resources (1, 2). LST often causes great suffering to patients, reduces the quality of life, and incurs high medical costs (3, 4). Continuation of life-sustaining treatment for critically ill patients is sometimes ill advised, while abandonment of life-sustaining treatment has legal and ethical implications (5, 6). The cardiac surgery intensive care unit (ICU) mainly treats patients who need intensive monitoring and treatment after cardiac surgery or major vascular surgery, especially those with organ failure, to preserve life through focused and intensive treatment, to gain time for primary treatment and to stabilize or improve physical condition. Since ICU patients are critically ill or even unconscious, many medical decisions are made by clinical physicians who inform family members. The final decision is made by family members, but the family members have different perceptions and attitudes toward life-sustaining treatment, and the family members’ decisions do not necessarily represent those of the patient (7). The purpose of this study was to investigate the attitudes of cardiac surgery ICU patients and their families toward LST and the consistency of the patient's and family's attitudes toward LST to provide a basis for more respect for the patient, patient autonomy in decision-making and more appropriate patient care.

## 2. Materials and methods

### 2.1. Subjects

Pre-cardiothoracic surgery Patients who will transfer to the ICU after cardiothoracic surgery in Nanjing First Hospital from May 2020 to December 2021 were selected as the survey population. Patient inclusion criteria were as follows: age >18 years; awake patients; good communication skills; normal mental status. Patient exclusion criteria were as follows: previous involvement in research related to LST, patients who could not cooperate with this study for various reasons. The inclusion criteria for family members were as follows: age >18 years; good communication skills; and primary caregiver of the patient. The exclusion criteria for family members were as follows: unstable mental state, family members who could not cooperate with this study for various reasons were excluded. Pairs of patients and family members who met the inclusion criteria of this study were eligible. After the informed consent of the department of cardiac surgery ICU, with the assistance of the medical staff of the department, the investigators used unified instructions to explain the purpose and significance of the study and obtain the informed consent from the patients and family members.

### 2.2. Tools

#### 2.2.1. General demographic information questionnaire

This tool, designed by the investigator, included the following questions: patient age, gender, marital status, education level, occupation, religion, medical payment method, and primary caregiver-patient relationship. Through literature review, all demographic factors that may influence attitudes towards LST were included.

#### 2.2.2. Patients’ willingness to care for life-sustaining treatment questionnaire

Refer to the End-of-Life Preferences Interview in the Borreani et al. (8) study and also according to Chinese cardiac surgery and ICU nursing experts. The end-of-life care willingness questionnaire was “During our assistance to you, it could be necessary to use technical tools, like a catheter or a nasogastric tube. If your physical condition requires it, would it be difficult for you to accept some of these technical tools?” consultation to revise the willingness to provide life-sustaining treatment entry to “If your condition changes and you experience cardiac arrest, would you be willing to receive cardiopulmonary resuscitation, electric defibrillation, pacemaker, nasal feeding, intra-aortic balloon counterpulsation, blood transfusion, noninvasive mechanical ventilation, invasive mechanical ventilation, hemodialysis, extracorporeal membrane oxygenation?” An explanation of each life-sustaining treatment was given, including the purpose of the operation, the method of operation, complications, and costs. In this study, 30 patients were selected for the pretest of this questionnaire, which had a Cronbach's alpha coefficient of 0.846.

#### 2.2.3. Willingness of family members for the patient to receive life-sustaining treatment care questionnaire

Similar to the willingness of patients to receive life-sustaining treatment care questionnaire, some of the entries were revised to use the question, “If your family member's condition changes and he or she goes into cardiac arrest, would you be willing for him or her to receive cardiopulmonary resuscitation, electric defibrillation, pacemaker, nasal feeding, intra-aortic balloon counterpulsation, blood transfusion, noninvasive mechanical ventilation, invasive mechanical ventilation hemodialysis, extracorporeal membrane oxygenation?” An explanation of each life support treatment, including the purpose of operation, operation method, complications, and costs was included. In this study, 30 family members were selected for the pretest of this questionnaire, and the Cronbach's alpha coefficient of this questionnaire was 0.824.

### 2.3. Data collection

All investigators involved in the survey received uniform training (The training includes the role of various LST, the possible complications, and the costs incurred by the program.), to avoid bias in administering the questionnaire. In the investigation, the patient and their family members were required to fill in the questions separately, and accompanied by the investigator throughout the whole process. To explain the questions that the respondents did not understand, but do not give induced answers, and inform the patient and their family members that their answers had no impact on the treatment of the disease. A total of 200 questionnaires were distributed, and 172 valid questionnaires were returned for a recovery rate of 86%; 15 patients refused to participate in the survey, and 13 recovered questionnaires were not qualified.

### 2.4. Statistical analysis

Data were double-checked for entry, and SPSS 19.0 was used for descriptive analysis of general data, with measurement data expressed as the mean ± standard deviation (*x* ± *s*) and count data described as frequency and percentage; the consistency of ICU patients’ and families’ attitudes toward life-sustaining treatment was tested by chi-square test with paired design, data not conforming to the chi-square test used the Fisher test, and *P* < 0.05 was considered statistically significant.

## 3. Results

### 3.1. General demographic data

Ages 29–79 with a mean age of 61.76 ± 11.608; gender: 97 (56.4%) males, 75 (43.6%) females; marital status: 151 (87.8%) married, 6 (3.5%) unmarried, 15 (8.7%) widowed; educational level: elementary school and less 56 (32.6%), junior high school 45 (26.2%), high school 42 (24.4%), university 27 (15.7%), master's and more 2 (1.2%); occupation: farmers 63 (36.6%), workers 20 (11.6%), business 8 (4.7%), institutions 14 (8.1%), retired 67 (39.0%); religious beliefs: Buddhism 2 (1.2%), Christianity 9 (5.2%), no religious beliefs 161 (93.6%); medical cost payment: self-pay 19 (11.0%), citizens’ medical insurance 93 (54.1%), new agricultural cooperative 60 (34.9%). Patient surgery: mitral valve replacement in 57 cases (33.1%), aortic valve replacement in 41 cases (23.8%), double valve replacement in 16 cases (9.3%), coronary artery bypass grafting in 40 cases (23.3%), and endoluminal isolation of aortic aneurysm in 18 cases (10.5%). Primary caregiver-patient relationships: spouse 79 (45.9%), son 38 (22.1%), daughter 42 (24.1%), and other relatives 13 (7.6%).

### 3.2. Attitudes of cardiac surgery ICU patients toward life-sustaining treatment

Table 1 The results of this study showed that the percentage of cardiac surgery ICU patients accepting various life-sustaining treatments ranged from 16.9% to 74.4%, among which the acceptance of noninvasive mechanical ventilation was highest, with 79.1% of patients willing to receive the treatment, only 40.7% of patients willing to receive invasive mechanical ventilation. The acceptance of electric defibrillation was the lowest, with only 16.9% of patients willing to receive the treatment, and only 20% of the patients are willing to receive cardiopulmonary resuscitation. Patients were usually unconscious when receiving electrical defibrillation, but in the results of this investigation, electrical defibrillation was the most unacceptable treatment for awake patients, which may be because the patient thinks that electrical defibrillation has a poor prognosis. The percentage of cardiac surgery ICU patients who were not willing to receive life-sustaining treatment ranged from 5.8% to 48.3%, and the least popular treatment was intra-aortic balloon counterpulsation (48.3%). A total of 8.1%–65.7% of patients did not think about receiving life-sustaining treatment, and the greatest percentage had not thought about electrical defibrillation (65.7%).

**Table 1:** Attitudes of cardiac surgery ICU patients toward life-sustaining treatments.

	YES [*n* (%)]	NO [*n* (%)]	Never thought about it [*n* (%)]
Cardiopulmonary resuscitation	36 (20.9)	52 (30.2)	84 (48.8)
Electric defibrillation	29 (16.9)	30 (17.4)	113 (65.7)
Pacemaker	110 (64.0)	45 (26.2)	17 (9.9)
Nasal feeding	128 (74.4)	30 (17.4)	14 (8.1)
Intra-aortic balloon counterpulsation	61 (35.5)	83 (48.3)	28 (16.3)
Blood transfusion	103 (59.9)	69 (40.1)	0 (0)
Noninvasive mechanical ventilation	136 (79.1)	10 (5.8)	26 (15.1)
Invasive mechanical ventilation	70 (40.7)	32 (18.6)	70 (40.7)
Hemodialysis	59 (34.3)	23 (13.4)	90 (52.3)
Extracorporeal membrane oxygenation	57 (33.1)	18 (10.5)	97 (56.4)

**Table 2:** Attitudes of family members of cardiac surgery ICU patients toward life-sustaining treatment.

	YES [*n* (%)]	NO [*n* (%)]	Never thought about it [*n* (%)]
Cardiopulmonary resuscitation	131 (76.2)	17 (9.9)	24 (14.0)
Electric defibrillation	117 (68.0)	21 (12.2)	34 (19.8)
Pacemaker	108 (62.8)	43 (25.0)	21 (12.2)
Nasal feeding	120 (69.8)	32 (18.6)	20 (11.6)
Intra-aortic balloon counterpulsation	112 (65.1)	41 (23.8)	19 (11.0)
Blood transfusion	121 (70.3)	36 (20.9)	15 (8.7)
Noninvasive mechanical ventilation	128 (74.4)	37 (21.5)	7 (4.1)
Invasive mechanical ventilation	87 (50.6)	21 (12.2)	64 (37.2)
Hemodialysis	93 (54.1)	18 (10.5)	61 (35.5)
Extracorporeal membrane oxygenation	69 (40.1)	22 (12.8)	81 (47.1)

**Table 3:** Consistency in attitudes of cardiac surgery ICU patients and family members toward patients receiving life-sustaining treatments.

	Patients [*n* (%)]	Family members [*n* (%)]				Kappa-value	95% CI	Chi-square test (*X*^2^)	*p*-value
		YES	NO	Never thought about it	Total				
Cardiopulmonary resuscitation	YES	32 (18.6)	1 (0.6)	3 (1.7)	36 (1.7)	0.147	0.101–0.190	23.048	0.000
	NO	40 (23.3)	11 (6.4)	1 (0.6)	52 (30.2)				
	Never thought about it	59 (34.3)	5 (2.9)	20 (11.6)	84 (48.8)				
	Total	131 (76.2)	17 (9.9)	24 (14.0)	172 (100)				
Electric defibrillation	YES	22 (12.8)	4 (2.3)	3 (1.7)	29 (16.9)	0.081	0.039–0.124	7.418	0.115
	NO	19 (11.0)	7 (4.1)	4 (2.3)	30 (17.4)				
	Never thought about it	76 (44.2)	10 (5.8)	27 (15.7)	113 (65.7)				
	Total	117 (68.0)	21 (12.2)	34 (19.8)	173 (100)				
Pacemaker	YES	80 (46.5)	15 (8.7)	15 (8.7)	110 (64.0)	0.252	0.212–0.279	28.142	0.000
	NO	16 (9.3)	24 (14.0)	5 (2.9)	45 (26.2)				
	Never thought about it	12 (7.0)	4 (2.3)	1 (0.6)	17 (9.9)				
	Total	108 (62.8)	43 (25.0)	21 (12.2)	172 (100)				
Nasal feeding	YES	98 (57.0)	18 (10.5)	12 (7.0)	128 (74.4)	0.218	0.164–0.249	20.435	0.000
	NO	11 (6.4)	13 (7.6)	6 (3.5)	30 (17.4)				
	Never thought about it	11 (6.4)	1 (0.6)	2 (1.2)	14 (8.1)				
	Total	120 (69.8)	32 (18.6)	20 (11.6)	172 (100)				
Intra-aortic balloon counterpulsation	YES	43 (25.0)	8 (4.7)	10 (5.8)	61 (35.5)	0.077	0.036–0.108	7.688	0.104
	NO	52 (30.2)	25 (14.5)	6 (3.5)	83 (48.3)				
	Never thought about it	17 (9.9)	8 (4.7)	3 (1.7)	28 (16.3)				
	Total	112 (65.1)	41 (23.8)	19 (11.0)	172 (100)				
Blood transfusion	YES	90 (52.3)	12 (7.0)	10 (5.8)	103 (59.9)	0.408	0.362–0.452	35.767	0.000
	NO	31 (18.0)	24 (13.9)	5 (2.9)	69 (40.1)				
	Total	121 (70.3)	36 (26.7)	15 (2.9)	172 (100)				
Noninvasive mechanical ventilation	YES	104 (60.5)	26 (15.1)	6 (3.5)	136 (79.1)	0.038	0.012–0.081	3.596	0.463
	NO	8 (4.7)	2 (1.2)	0 (0)	10 (5.8)				
	Never thought about it	16 (9.3)	9 (5.2)	1 (0.6)	26 (15.1)				
	Total	128 (74.4)	37 (21.5)	7 (4.1)	172 (100)				
Invasive mechanical ventilation	YES	61 (35.5)	0 (0)	9 (5.2)	70 (40.7)	0.597	0.526–0.645	123.503	0.000
	NO	15 (8.7)	15 (8.7)	2 (1.2)	32 (18.6)				
	Never thought about it	11 (6.4)	6 (3.5)	53 (30.8)	70 (40.7)				
	Total	87 (50.6)	21 (12.2)	64 (37.2)	172 (100)				
Hemodialysis	YES	53 (30.8)	2 (1.2)	4 (2.3)	59 (34.3)	0.404	0.358–0.442	55.681	0.000
	NO	8 (4.7)	7 (4.1)	8 (4.7)	23 (13.4)				
	Never thought about it	32 (18.6)	9 (5.2)	49 (28.5)	90 (52.3)				
	Total	93 (54.1)	18 (10.5)	61 (35.5)	172 (100)				
Extracorporeal membrane oxygenation	YES	44 (25.6)	5 (2.9)	8 (4.7)	57 (33.1)	0.456	0.421–0.482	60.648	0.000
	NO	6 (3.5)	6 (3.5)	6 (3.5)	18 (10.5)				
	Never thought about it	19 (11.0)	11 (6.4)	67 (39.0)	97 (56.4)				
	Total	69 (40.1)	22 (12.8)	81 (47.1)	172 (100)				

Data are presented as *n* (%) unless otherwise indicated; the *p*-value was determined by the chi-square test for multiple comparisons, the Kappa-value was determined by chi-square test with paired design for the consistency of attitudes.

### 3.3. Attitudes of family members of cardiac surgery ICU patients toward life-sustaining treatments

Table 2 the results of this study showed that the percentage of family members of cardiac surgery ICU patients who expressed attitudes toward patients receiving various life-sustaining treatment treatments ranged from 40.1% to 76.2%, with the greatest percentage favoring cardiopulmonary resuscitation (76.2%) and the lowest percentage approving extracorporeal membrane oxygenation (40.1%). The percentage of family members who were unwilling to have patients receive life-sustaining treatment ranged from 9.9% to 25.0%. The life-sustaining treatment that families were least willing for patients to receive was a pacemaker (25.0%). A total of 2.9%–37.2% of family members had not thought about patients receiving life-sustaining treatment, and the least considered treatment was extracorporeal membrane oxygenation (47.1%). The results showed that the family members’ attitude towards LST was inconsistent with the patients, and the most willing LST and the least willing LST were different from the patients.

### 3.4. Consistency in attitudes of cardiac surgery ICU patients and family members toward patients receiving life-sustaining treatment

Table 3 the results of this study showed that the consistency of attitudes toward life-sustaining treatment among cardiac surgery ICU patients pre-operatively and family members ranged from 12.8% to 60.5% for willingness to accept LSTs, patients and family members the most willingness to accept was noninvasive mechanical ventilation (60.5%), 1.2% to 14.5% for unwilling, the most unwilling to accept was intra-aortic balloon counterpulsation (14.5%), and 0.6% to 39.0% for never thought of it, The most unthought of LSTs was extracorporeal membrane oxygenation (39%). The results of the consistency analysis showed that the kappa values ranged from 0.218 to 0.597 (*P* < 0.05). According to the consistency in attitudes (kappa values) ranked the life-sustaining treatment from highest to lowest are: ① invasive mechanical ventilation, ② extracorporeal membrane oxygenation, ③ blood transfusion, ④ hemodialysis, ⑤ pacemaker, ⑥ nasal feeding, ⑦cardiopulmonary resuscitation, ⑧ electric defibrillation, ⑨ intra-aortic balloon counterpulsation, ⑩ noninvasive mechanical ventilation. The results showed the cardiac surgery ICU patients and family members had poor consistency in their attitudes toward LSTs, with differences in attitudes toward cardiopulmonary resuscitation, pacemakers, nasal feeding, blood transfusions, invasive mechanical ventilation, hemodialysis, and extracorporeal membrane oxygenation (*P* < 0.05).

## 4. Discussion

The survey of cardiac surgery ICU patients’ attitudes toward life-sustaining treatment found that the most preferred life-sustaining treatment by patients was noninvasive mechanical ventilation (79.1%). The incidence of pulmonary dysfunction in cardiac surgery under extracorporeal circulation is as high as 20%–30% (9–11). In patients with severe respiratory failure, invasive mechanical ventilation, such as tracheal intubation or tracheotomy, is often required to improve airway patency; however, prolonged tracheal intubation or tracheotomy can often increase patient suffering, medical costs and the chance of ventilator-associated pneumonia, while treatment with noninvasive positive pressure ventilation improves patient survival and reduces complications, such as ICU-acquired infections (12, 13). Therefore, patients requiring mechanical ventilation after cardiac surgery may first consider the option of noninvasive mechanical ventilation. Without affecting the patient's prognosis, noninvasive mechanical ventilation is more cooperative. Patients were most reluctant to receive intra-aortic balloon counterpulsation (48.3%), which is increasingly used in patients with severe hemodynamic disorders and has become one of the most widely used cardiac assist devices and indispensable for the treatment of cardiac surgery ICU patients, but the application of this life-sustaining treatment is associated with a high number of complications, including limb ischemia, thrombosis, embolism, arterial entrapment, bleeding, infection and thrombocytopenia, with a complication rate of 15%, of which 11% are serious complications (14, 15). The use of intra-aortic balloon counterpulsation requires the patient to be bedridden with limb braking. This restricts the patient's movement, while concerns about disease prognosis predispose them to agitation. The most important life-sustaining treatment that patients did not think about was electric defibrillation (65.7%). Compared to other life-sustaining treatments, electric defibrillation was a treatment that patients were more aware of, but most of them learned about electric defibrillation from media such as television shows and thought that the procedure was the most end-of-life resuscitation measure, so they had not imagined that they would need it.

Family members of cardiac surgery ICU patients had more positive attitudes toward patients receiving various life-sustaining treatments than patients, and the most popular treatment was cardiopulmonary resuscitation (76.2%), which may be considered by family members as one of the most effective measures to save patients’ lives. The life-sustaining treatment that the family members were least willing for the patient to receive was a pacemaker (25.0%). An artificial pacemaker uses a specific frequency of pulsed electric current to stimulate the heart through electrodes that regulate its rhythm. The study found decreases in mood, dyspnea, and lower scores in quality of life scores among pacemaker recipients compared to prepacemaker scores, and a deterioration in function, discomfort, and quality of life the longer the pacemaker was implanted (16, 17). There are many complications associated with the use of pacemakers, so these are probably the reasons why patients’ families were most reluctant for patients to receive pacemakers. The life-sustaining treatment that family members had thought about least was extracorporeal membrane oxygenation (47.1%). This life-sustaining treatment is relatively new and expensive, and many family members reported that they had not heard of this technique and therefore had not thought about patients receiving it. For nasal feeding and blood transfusion, family acceptance was higher, mainly because these two operations are less harmful to the patient, less expensive, and better known.

The results of this study showed that the consistency of attitudes between cardiac surgery ICU patients and their family members toward life-sustaining treatment was average, with kappa values ranging from 0.218 to 0.597, indicating that medical decisions made by family representatives do not adequately reflect patients’ wishes. The consistency of patients’ and family members’ attitudes toward life-sustaining ranged from 12.8% to 60.5% for willingness to have LSTs, 1.2% to 19.8% for unwilling, and 0.6% to 39.0% for never thought about it. In China, despite the increasing emphasis on patient autonomy, because most cardiac surgery ICU patients are elderly patients with an average age of 61.76 ± 11.608 and low literacy level, with 32.6% having elementary school education or less, patients are less informed about medical developments and various life-sustaining treatments. Family members take the main responsibility for communication with physicians and medical decision-making, and physicians also rely more on family members to make decisions. In traditional Asian and Chinese cultures, it is taboo to discuss death with the patient himself, so the decision to give up or insist on life-sustaining treatment at the end of life is mostly made by family members (18). In our ICU, 78.10% of the patients who gave up treatment did so at the suggestion of their family members, while only one case was decided by the patient. In 15.33% of the cases, the recommendation to discontinue LSTs was made by the ICU bedside doctors, and 9.49% of the recommendations were made by the doctors in charge of the original department together with the patient's family members. There was no case in which the ICU bedside doctor and the doctor in charge of the original department independently made the recommendation. For family members, the main factors to consider when stopping life-sustaining treatments are economic and moral factors. Traditional Chinese concepts make it difficult for most family members to actively decide to stop treatment. Family members always want to save the patient's life at all costs, even if they have already spent all they have. The decision to give up on treatment can lead to condemnation from relatives and friends. What critically ill patients want more than anything else is the care and companionship of their family members, and they want treatment that improves their quality of life and reduces their suffering (19). They do not want to burden their families financially and psychologically because of their illness, and they want to be able to make their own decisions about whether to hold on or give up life support at the end of their lives (20). Therefore, having family members make patients’ medical decisions does not reflect patients’ wishes and is not conducive to respecting patients’ autonomy. The right to informed consent should primarily be enjoyed by patients, but the reality in China makes it impossible not to value the participation of patients’ families; however, the extent to which patients’ relatives can represent patients’ interests should be examined. In most cases, relatives represent the patient's interests, but there are cases where interests do not coincide, and even when they do, there are often differences in values that may lead to inconsistent choices between the patient and the family. It is important to establish preemptive care in life support treatment decisions for ICU patients to be more respectful of patients’ rights, to reduce the psychological burden on families and to help physicians make decisions (21–23).

It is recommended that medical staff encourage family members to communicate with patients about their willingness to use life support and encourage patients to participate in medical decision-making. Medical staff should generally allow families to make choices if they are fully informed. When patients and their relatives disagree, medical staff should first consider and respect the patient's opinion and give the patient or family advice on whether to use life support based on the patient's condition, the family's economic condition, and values. In recent years, advance directives have been gaining attention in China, and the formulation of advance directives is a shortcut to help patients express their terminal treatment intentions, and with the support and respect of families, it is easier to be implemented in ICU patients. It is recommended that medical staff disseminate knowledge and importance of advance directive to ICU patients and their families, and explore solutions to the family-based decision-making model.

## 5. Conclusion

The results of this study showed that the consistency of attitudes towards life-sustaining treatment was weak between cardiac surgery ICU patients and family members, and the family choice of life-sustaining treatment was not representative of patient decision. Due to the limitation of time, manpower and material resources, this study used convenient sampling, which only represents the consistency of cardiac surgery ICU patients and their families towards LST. It is suggested that subsequent researchers increase the sample size to conduct cross-sectional survey, further explore the plan indicated in advance under the background of Chinese culture, and promote the communication and record the patients’ willingness to accept LST. To improve the consistency of attitudes of ICU patients and their families towards LST and help improve the quality of terminal care for patients.

## Data Availability

The original contributions presented in the study are included in the article/**Supplementary Material**, further inquiries can be directed to the corresponding author.

## References

[1] American Thoracic Society. Withholding and withdrawing life-sustaining therapy. Ann Intern Med. (1991) 115:478–85. 10.7326/0003-4819-115-6-478 11642893

[2] Berlinger N Jennings B Wolf SM. The hastings center guidelines for decisions on life-sustaining treatment and care near the End of life. New York: Oxford University Press (2013).

[3] Chavez G Richman IB Kaimal R Bentley J Yasukawa LA Altman RB Reversals and limitations on high-intensity, life-sustaining treatments. PLoS One. (2018) 13(2):e0190569. 10.1371/journal.pone.0190569 29489814 PMC5830043

[4] Sunjung K Sunghee HT. Family members’ knowledge and attitude toward life-sustaining treatment decisions for patients in the intensive care unit. J Hosp Palliat Nurs. (2021) 23(3):256–63. 10.1097/NJH.0000000000000750 33840799 PMC8104012

[5] Alexander C Edward RK Annabel L. Withdrawing life-sustaining treatment: a stock-take of the legal and ethical position. J Med Ethics. (2019) 45(12):794–9. 10.1136/medethics-2019-105599 31488520

[6] Kirsch RE Balit CR Carnevale FA Latour JM Larcher V. Ethical, cultural, social, and individual considerations prior to transition to limitation or withdrawal of life-sustaining therapie. Pediatr Crit Care Med. (2018) 19(8S Suppl 2):S10–S18. 10.1097/PCC.0000000000001488. 30080802

[7] Shalowitz DI Garrett-Mayer E Wendler D. The accuracy of surrogate decision maker: a systematic review. Arch Intern Med. (2006) 166(5):493–7. 10.1001/archinte.166.5.493 16534034

[8] Borreani C Bmnelli C Miccinesi G Morino P Piazza M Piva L Eliciting individual preferences about death:development of the end of life preferences interview. J Pain Symptom Manage. (2008) 36(4):335–50. 10.1016/j.jpainsymman.2007.10.013 18440766

[9] Luc JGY Aboelnazar NS Himmat S Hatami S Haromy A Matsumura N A leukocyte filter does not provide further benefit during ex vivo lung perfusion. ASAIO J. (2017) 63:672–8. 10.1097/MAT.0000000000000550. 28234641

[10] Ziyaeifard M Alizadehasl A Massoumi G. Modified ultrafiltration during cardiopulmonary bypass and postoperative course of pediatric cardiac surgery. Res Cardiovasc Med. (2014) 3:e17830. 10.5812/cardiovascmed.17830 25478538 PMC4253790

[11] Hofmann B Kaufmann C Stiller M Neitzel T Wienke A Silber RE Positive impact of retrograde autologous priming in adult patients undergoing cardiac surgery: a randomized clinical trial. J Cardiothorac Surg. (2018) 13:50. 10.1186/s13019-018-0739-0 29784004 PMC5963082

[12] Brochard L. Mechanical ventilation: invasive versus noninvasive. Eur Respir J Suppl. (2003) 47:31–7. 10.1183/09031936.03.00050403 14621115

[13] Thomas BL Florence A. Non-invasive mechanical ventilation to prevent ICU-acquired infection. Infect Disord Drug Targets. (2011) 11(4):384–8. 10.2174/187152611796504782 21679143

[14] Davidson J Baumgariner F Omari B Milliken J. Intra-aortic balloon pump: indications and complications. J Natl Med Assoc. (1998) 90(3):137–40. PMCID: 9549976 PMC2608336

[15] Cohen M Dawson MS Kopistansky C McBride R. Sex and other predictors of intra-aortic balloon counterpulsation 2 related complications:prospective study of 1119 consective patients. Am Heart J. (2000) 139(2):282–7. 10.1067/mhj.2000.101489 10650301

[16] Barros RT Carvalho SM Silva MA Borges JB. Evaluation of patients’ quality of life aspects after cardiac pacemaker implantation. Rev Bras Cir Cardiovasc. (2014) 29(1):37–44. 10.5935/1678-9741.20140009 24896161 PMC4389479

[17] van Eck JW van Hemel NM Kelder JC van den Bos AA Taks W Grobbee DE Poor health-related quality of life of patients with indication for chronic cardiac pacemaker therapy. Pacing Clin Electrophysiol. (2008) 31(4):480–6. 10.1111/j.1540-8159.2008.01018.x 18373768

[18] Kim SH. Family surrogates’ decision regret and psychological stress about end-of-life cancer treatments: path analysis. J Korean Acad Nurs. (2018) 48:578–87. 10.4040/jkan.2018.48.5.578 30396195

[19] Sharon R Andrew BC Martin MK. Withdrawing life-sustaining treatment: ethical considerations. Surg Clin North Am. (2007) 87(4):919–36. 10.1016/j.suc.2007.07.013 17888789

[20] Mirinae K Minju K. We want more than life-sustaining treatment during end-of-life care: focus-group interviews. Int J Environ Res Public Health. (2021) 18(9):4415. 10.3390/ijerph18094415 33919357 PMC8122594

[21] Sudore RL Heyland DK Lum HD Rietjens AC Korfage IJ Ritchie CS Outcomes that define successful advance care planning: a delphi panel consensus. J Pain Symptom Manag. (2018) 55:245–55. 10.1016/j.jpainsymman.2017.08.025 PMC5794507 28865870

[22] Hamayoshi M Goto S Matsuoka C Kono A Miwa K Tanizawa K Effects of an advance care planning educational programme intervention on the end-of-life care attitudes of multidisciplinary practitioners at an acute hospital: a pre- and post-study. Palliat Med. (2019) 33:1158–65. 10.1177/0269216319860707 31257989

[23] Jimenez G Tan WS Virk AK Low CK Car J Ho HY. Overview of systematic reviews of advance care planning: summary of evidence and global lessons. J Pain Symptom Manag. (2018) 56:436–59. 10.1016/j.jpainsymman.2018.05.016 29807158

