# Supplementary material for: The attitudes about life-sustaining treatment among cardiac surgery ICU patients and their families
Source: Front Surg. 2023 May 19;10:1079337. doi: 10.3389/fsurg.2023.1079337 (PMC10235522; doi:10.3389/fsurg.2023.1079337)
Supplement: Supplementary file 1 [file Datasheet1.pdf]

supplement file

Patients' willingness to care for life-sustaining treatment questionnaire

Questionnaire instructions: This questionnaire is only used to investigate your attitude towards life support treatment survey, it will not affect the choice of life support treatment by doctors, please fill out the questionnaire honestly and keep the information you fill confidential.Thank you very much for your support with this study.

1. If your condition changes and you experience cardiac arrest, would you be willing to receive cardiopulmonary resuscitation?

a.YES b.NO c.Never thought about it

2. If your condition changes and you experience cardiac arrest, would you be willing to receive electric defibrillation?

a.YES b.NO c.Never thought about it

3. If your condition changes and you experience cardiac arrest, would you be willing to receive pacemaker?

a. YES b.NO c.Never thought about it

4. If your condition changes and you experience cardiac arrest, would you be willing to receive nasal feeding?

a. YES b.NO c.Never thought about it

5. If your condition changes and you experience cardiac arrest, would you be willing to receive intra-aortic balloon counterpulsation?

a. YES b.NO c.Never thought about it

6. If your condition changes and you experience cardiac arrest, would you be willing to receive blood transfusion?

a.YES b.NO c.Never thought about it

7. If your condition changes and you experience cardiac arrest, would you be willing to receive noninvasive mechanical ventilation?

a.YES b.NO c.Never thought about it

8. If your condition changes and you experience cardiac arrest, would you be willing to receive invasive mechanical ventilation?

a. YES b.NO c.Never thought about it

9. If your condition changes and you experience cardiac arrest, would you be willing to receive hemodialysis?

a. YES b.NO c.Never thought about it

10. If your condition changes and you experience cardiac arrest, would you be willing to receive extracorporeal membrane oxygenation?

a.YES b.NO c.Never thought about it

Willingness of family members for the patient to receive life-sustaining treatment care questionnaire

Questionnaire instructions: This questionnaire is only used to investigate your attitude towards life support treatment survey, it will not affect the choice of life support treatment by doctors, please fill out the questionnaire honestly and keep the information you fill confidential.Thank you very much for your support with this study.

1.If your family member's condition changes and he or she goes into cardiac arrest, would you be willing for him or her to receive cardiopulmonary resuscitation?

a.YES b.NO c.Never thought about it

2.If your family member's condition changes and he or she goes into cardiac arrest, would you be willing for him or her to receive electric defibrillation?

a.YES b.NO c.Never thought about it

3.If your family member's condition changes and he or she goes into cardiac arrest, would you be willing for him or her to receive pacemaker?

b. YES b.NO c.Never thought about it

4.If your family member's condition changes and he or she goes into cardiac arrest, would you be willing for him or her to receive nasal feeding?

b. YES b.NO c.Never thought about it

5.If your family member's condition changes and he or she goes into cardiac arrest, would you be willing for him or her to receive intra-aortic balloon counterpulsation?

b. YES b.NO c.Never thought about it

6.If your family member's condition changes and he or she goes into cardiac arrest, would you be willing for him or her to receive blood transfusion?

a.YES b.NO c.Never thought about it

7.If your family member's condition changes and he or she goes into cardiac arrest, would you be willing for him or her to receive noninvasive mechanical ventilation?

a.YES b.NO c.Never thought about it

8.If your family member's condition changes and he or she goes into cardiac arrest, would you be willing for him or her to receive invasive mechanical ventilation?

a.YES b.NO c.Never thought about it

9.If your family member's condition changes and he or she goes into cardiac arrest, would you be willing for him or her to receive hemodialysis?

a.YES b.NO c.Never thought about it

10.If your family member's condition changes and he or she goes into cardiac arrest, would you be willing for him or her to receive extracorporeal membrane oxygenation?

a.YES b.NO c.Never thought about it
